# Perinatal quality-of-life trajectories and determinants among high-risk pregnant women and their husbands: a prospective cohort study

**DOI:** 10.4069/whn.2026.08.21

**Published:** 2026-09-29

**Authors:** Hyunjin Cho, Sukhee Ahn

**Affiliations:** 1 College of Nursing, Chodang University, Muan, Korea; 2 College of Nursing, Chungnam National University, Daejeon, Korea

**Keywords:** Anxiety, High-risk pregnancy, Quality of life, Social support

## Abstract

**Purpose:**

This study examined changes in quality of life (QoL) among high-risk pregnant women and their husbands across the perinatal period and identified physical and psychosocial factors associated with these changes over time.

**Methods:**

A total of 149 couples experiencing high-risk pregnancies were recruited from an online community for mothers in South Korea and followed from pregnancy (T1) to 6 weeks postpartum (T2) and 12 weeks postpartum (T3). QoL was measured using the World Health Organization Quality of Life-BREF, and anxiety, fatigue, perceived social support, and marital adjustment were assessed using validated self-report instruments. A linear mixed model was used to examine changes in QoL over time, and Pearson correlation and hierarchical multiple regression analyses were used to identify predictors of QoL at each time point.

**Results:**

Both pregnant women and their husbands had significant declines in physical and psychological QoL from pregnancy to 6 weeks postpartum, followed by partial recovery by 12 weeks postpartum. Husbands reported consistently higher QoL scores than their wives at all time points. Higher anxiety and lower perceived social support were the most consistent predictors of lower QoL in both partners; maternal QoL was also associated with fatigue, whereas husbands’ QoL was more strongly associated with marital satisfaction and parenting-related stress after childbirth. The regression models explained 33% to 66% of the variance in QoL across the three time points.

**Conclusion:**

These findings underscore the interdependence of QoL among high-risk pregnant women and their husbands throughout the perinatal period. Nursing interventions that address anxiety, fatigue, social support, and couple adaptation may improve family well-being and support recovery after a high-risk pregnancy.

## Introduction

As of 2025, the mean age of primiparous women was 33.8 years, and mothers of advanced maternal age (35 years and older) accounted for 37.3% of all births [1]. Increases in maternal age and delayed pregnancy have contributed to more high-risk pregnancies, including gestational diabetes mellitus (GDM), gestational hypertension, and preterm labor, thereby increasing the risk of preterm birth. Since 2014, the proportion of preterm infants has increased approximately 1.5-fold; by 2024, about 10% of all newborns were born preterm (less than 37 weeks of gestation), increasing the burden associated with high-risk pregnancies involving preterm and low-birth-weight infants [2]. Since 2018, the proportion of high-risk mothers in South Korea has also increased. Mothers of advanced maternal age accounted for 31.8% of all births in 2018, 36.3% in 2023, 35.9% in 2024, and 37.3% in 2025 [1]. These demographic shifts indicate a growing need for risk management throughout pregnancy and childbirth.

A high-risk pregnancy is a condition in which pregnancy-related complications or preexisting chronic diseases threaten the health of the pregnant woman or fetus [3]. It is therefore a complex health issue requiring psychosocial support in addition to medical management and physical treatment. In particular, 92.2% of pregnant women diagnosed with preterm labor require hospitalization. Even among those receiving outpatient care, medication and continuous monitoring place substantial constraints on family life [4]. Thus, high-risk pregnancy extends beyond the woman’s individual experience and affects the entire family [5].

Quality of life (QoL) is a multidimensional concept encompassing physical, emotional, and social well-being. Health-related QoL is particularly useful for evaluating how illness, treatment, and recovery affect an individual’s life [6]. Pregnancy is a major life event that affects women’s overall well-being and involves complex psychosocial factors, such as uncertainty and role transition, in addition to physiological changes [7]. In high-risk pregnancy, hospitalization, treatment, persistent anxiety about fetal health, and restrictions on daily activities may further reduce women’s QoL [8]. Pregnant women may experience physical and psychological discomfort related to treatment and management, while their husbands may face financial strain, anxiety about pregnancy outcomes, and emotional stress. High-risk pregnancy may therefore affect QoL in both partners [8]. During the transition to parenthood, QoL in pregnant women and their husbands, whether in low- or high-risk pregnancies, should be understood not as a fixed state but as a trajectory that changes over time [2,5].

The QoL of pregnant women is shaped by psychosocial factors as well as physical changes. Previous studies have reported that physical symptoms, fatigue, depression, and anxiety influence women’s daily functioning and emotional well-being throughout pregnancy [8,9]. Among pregnant women with high-risk pregnancies, higher levels of depression and anxiety are associated with lower emotional stability and more difficulty with daily functioning, while fatigue and physical symptoms intensify physical discomfort; together, these factors are associated with lower QoL [9-12]. In contrast, parenting confidence, maternal identity, and maternal-fetal attachment facilitate psychological adaptation and improve QoL. Spousal and social support also help reduce depression and anxiety and thereby contribute to better QoL [9-12]. Although pregnant women’s QoL is closely related not only to individual internal factors but also to family interactions and social support systems, previous research has not sufficiently examined QoL trajectories and their determinants for both pregnant women and husbands within pregnancy and childbirth as a family-level event.

Although high-risk pregnancy is a complex health issue that requires emotional support and comprehensive family-centered interventions, existing research has several important limitations. First, studies of pregnant women with high-risk conditions have mostly focused on short-term outcomes within 6 weeks postpartum [13], leaving longitudinal changes from pregnancy through postpartum recovery insufficiently examined. This cross-sectional or short-term approach limits understanding of how high-risk pregnancy affects pregnant women and their husbands over time. Second, because most studies have centered on pregnant women’s experiences [8-10,12,13], less is known about couple-level QoL and adaptation processes that include husbands [5,14]. High-risk pregnancy can cause substantial psychological burden and stress for husbands as well as pregnant women [5,14], and dyadic adaptation may influence health outcomes [14], but couple-level adaptation has rarely been examined comprehensively. Third, few longitudinal studies have systematically tracked and compared how factors associated with QoL change across the antenatal and postpartum periods, especially among couples experiencing high-risk pregnancies.

As family-centered nursing receives increasing attention, it is important to recognize that pregnant women and their husbands face different challenges and need different resources during the antenatal and postpartum recovery periods. Identifying key predictors at each time point can provide a basis for developing tailored interventions [9-16]. Prior findings that QoL-related factors differ between the antenatal and postpartum periods support the need for nursing interventions that reflect the characteristics of each period. Therefore, the theoretical basis for family-centered nursing interventions should be strengthened by considering antenatal and postpartum time points, systematically examining QoL-related factors in both pregnant women and their husbands, and clarifying the direction of each variable [9-16].

In summary, prior findings that factors associated with QoL vary between the antenatal and postpartum periods [9,10,12,13,17] suggest the need for nursing interventions tailored to each period. However, although high-risk pregnancy can substantially affect QoL in both pregnant women and their husbands, few longitudinal studies have followed couples from pregnancy through postpartum recovery. This study therefore longitudinally analyzed changes in QoL among pregnant women experiencing high-risk pregnancies and their husbands at three time points: 20–32 weeks of gestation, 6 weeks postpartum, and 12 weeks postpartum. It also examined physical and psychosocial factors associated with QoL at each time point. The findings may provide empirical evidence for developing tailored nursing interventions based on the time-specific characteristics and needs of couples experiencing high-risk pregnancy.

This study aimed to (1) identify QoL levels and changes among couples (pregnant women and their husbands) experiencing high-risk pregnancies at 20-32 weeks of gestation, 6 weeks postpartum, and 12 weeks postpartum; (2) determine antenatal QoL for each partner according to sociodemographic and obstetric characteristics; (3) analyze relationships between QoL and study variables by time point and partner; and (4) identify physical and psychosocial factors associated with QoL for each partner at each time point.

This study was grounded in Antonovsky’s salutogenic theory [18] to understand changes in QoL and factors associated with QoL among pregnant women with high-risk pregnancies and their husbands. Salutogenic theory explains that an individual’s position on the health-disease continuum is shaped by the interaction between stressors and generalized resistance resources (GRRs), which help individuals manage and overcome stressors, as well as by the sense of coherence (SOC) that develops through these experiences. In this context, stressors challenge psychological resources, and health outcomes are shaped less by the stressors themselves than by individuals’ cognitive appraisal and coping responses.

Pregnancy and childbirth are major life transitions that involve physical, psychological, and social changes for both pregnant women and their husbands. High-risk pregnancy, in particular, creates a complex stressful context because of perceived physical risk and uncertainty about fetal health [18]. During pregnancy, pregnancy stress, fatigue, and anxiety are major stressors; during the postpartum period, parenting stress becomes an additional major stressor.

In this study, social support and the spousal relationship were conceptualized as GRRs across both the antenatal and postpartum periods, and maternal and paternal role confidence were added as postpartum GRRs. Social support and the spousal relationship function as external GRRs by providing emotional support and practical assistance that help individuals perceive stressful situations as understandable and manageable [19]. Maternal and paternal role confidence function as internal GRRs by strengthening perceived control and self-efficacy in parental roles, thereby helping maintain and improve QoL [20,21].

On the basis of salutogenic theory, this study used stressors (pregnancy stress, fatigue, anxiety, and parenting stress) and GRRs (social support, spousal relationship, and maternal or paternal role confidence) as a framework for explaining QoL at each antenatal and postpartum time point (Figure 1).

## Methods

**Ethics statement:** This study was approved by the Institutional Review Board of Chungnam National University (202007-SB-094-01). Informed consent was obtained from the participants.

### Study design

This prospective cohort study examined changes in QoL and related factors among pregnant women experiencing high-risk pregnancies and their husbands from 20 to 32 weeks of gestation through 6 and 12 weeks postpartum. The study was reported according to the Strengthening the Reporting of Observational Studies in Epidemiology checklist [22].

### Participants

The study population consisted of pregnant women diagnosed with high-risk pregnancies and their husbands, recruited from an online community for mothers (“Mom cafe”), an online community for mothers in South Korea. Eligible participants were pregnant women at 20 to 32 weeks of gestation who had been diagnosed with a high-risk pregnancy and their husbands. In this study, high-risk pregnancy was defined as a medical condition diagnosed by healthcare professionals that could threaten the health of the pregnant woman or fetus [3]. Conditions requiring additional medical management included gestational hypertensive disorders, GDM, preterm labor, placenta previa, incompetent internal os of the cervix, multiple pregnancy, a history of recurrent miscarriage, and fetal growth restriction [4]. Pregnant women with these diagnoses and husbands who agreed to participate in all three survey rounds were included. Couples were excluded if the woman had not been diagnosed with a high-risk pregnancy, if cognitive impairment or severe psychiatric illness prevented questionnaire completion, or if couple-level data could not be obtained because of the dyadic study design (e.g., unmarried or divorced).

The sample size was calculated using G*Power 3.1 (Heinrich Heine University Düsseldorf, Düsseldorf, Germany). On the basis of a previous study [11] reporting that psychosocial factors, such as anxiety and social support, explained approximately 40% of the variance in health-related QoL among pregnant women (R^2^=0.40), the effect size for multiple regression analysis was set at a medium level (f^2^=.15). With a significance level of α=.05, power of 1–β=.80, and nine independent variables (women’s education, husbands’ education, history of hospitalization, fatigue, anxiety, stress [pregnancy stress in the antenatal period and parenting stress in the postpartum period], social support, spousal relationship, and maternal and paternal role confidence), the minimum required sample size was 114 participants. Allowing for an estimated 30% dropout rate from the antenatal to postpartum follow-up periods, the target sample at T1 was 170 to 200 couples (pregnant women and their husbands).

At T1 (20 to 32 weeks of gestation), 430 couples were initially recruited. Following screening for a high-risk pregnancy diagnosis, 200 couples met the eligibility criteria and were invited to participate, resulting in a final T1 analytic sample of 149 couples who completed the baseline survey. At T2 (6 weeks postpartum), due to loss to follow-up (n=15) and missing couple-level data due to husbands’ nonparticipation (n=12), the final analytic sample consisted of 122 couples. At T3 (12 weeks postpartum), loss to follow-up (n = 8) and missing couple-level data due to husbands’ nonparticipation (n = 6), the final analytic sample consisted of 108 couples. Thus, the analyses included 149 couples at T1, 122 at T2, and 108 at T3sss (Figure 2).

### Instruments

During the antenatal period (T1, 20 to 32 weeks of gestation), pregnancy stress, anxiety, and fatigue were measured as stressors, and social support and the spousal relationship were measured as GRRs. At 6 weeks postpartum (T2) and 12 weeks postpartum (T3), parenting stress, anxiety, and fatigue were assessed as stressors, and maternal and paternal role confidence were added as GRRs alongside social support and the spousal relationship. QoL was measured as the health outcome at all three time points (T1, T2, and T3). Before use, permission was obtained by email from the original authors or the developers of the Korean versions of all instruments.

#### Quality of life (T1, T2, T3)

QoL was measured using the World Health Organization Quality of Life-BREF, which was developed by the World Health Organization and translated and validated in Korean by Min et al. [23]. The instrument includes 26 items: four domains (physical health, seven items; psychological health, six items; social relationships, three items; environment, eight items) and two items on overall QoL and general health. Items are rated on a 5-point Likert scale. According to the scoring formula for the Korean version [23], each domain score was calculated by multiplying the mean raw score for that domain by 4. The total QoL score was calculated as the mean of all 26 items (sum divided by 26), with scores ranging from 1 to 5. Higher scores indicate better QoL. The two items on overall QoL and general health were presented separately. In Min et al. [23], the overall reliability was Cronbach’s α=.89, with domain reliabilities ranging from .58 to .77. In the present study, reliability for pregnant women was α=.90 at T1, α=.85 at T2, and α=.86 at T3; for husbands, reliability was α=.83 at T1, α=.89 at T2, and α=.91 at T3.

#### Pregnancy stress (T1)

Pregnancy stress was measured using the tool developed by Kim and Chung [24]. The instrument consists of 36 items across seven factors, rated on a 4-point Likert scale, with total scores ranging from 36 to 144. Higher scores indicate greater pregnancy stress. Although this tool was originally developed and validated only for pregnant women, it was applied to husbands without modification because the items cover domains that both partners may experience, including physical and psychological changes related to pregnancy, maternal and fetal health, and family support. Reliability at development was Cronbach’s α=.85 [24]. In this study, reliability at T1 was α=.88 for pregnant women and α=.89 for husbands.

#### Anxiety (T1, T2, T3)

Anxiety was assessed using a tool developed by Spielberger et al. [25] and translated into Korean by Kim and Shin [26]. It consists of 20 items rated on a 4-point Likert scale, with total scores ranging from 20 to 80. Higher scores indicate higher anxiety. The reliability of the original tool was Cronbach’s α=.84 [25], and reliability was .87 in Kim and Shin [26]. In the present study, reliability for pregnant women was α=.85 at T1, α=.89 at T2, and α=.91 at T3; for husbands, reliability was α=.86 at T1, α=.92 at T2, and α=.93 at T3.

#### Fatigue (T1, T2, T3)

Fatigue was measured using the shortened 10-item fatigue scale, a simplified version of the Fatigue Symptom Checklist developed by Milligan et al. [27] and translated into Korean by Song [28]. The instrument used in this study consists of nine items rated on a 4-point Likert scale, with total scores ranging from 10 to 40. Higher scores indicate greater fatigue. Reliability was Cronbach’s α=.87 for the original tool [27] and .87 in Song [28]. In this study, reliability for pregnant women was α=.85 at T1, α=.87 at T2, and α=.90 at T3; for husbands, reliability was α=.84 at T1, α=.93 at T2, and α=.94 at T3.

#### Relationship with spouse (T1, T2, T3)

The spousal relationship was measured using the Dyadic Adjustment Scale developed by Spanier [29] and translated into Korean by Cho et al. [30]. It consists of 10 items, with total scores ranging from 0 to 51. Higher scores indicate greater satisfaction with the spousal relationship. The reliability of the Korean version was Cronbach’s α=.92 [30]. In this study, reliability for pregnant women was α=.81 at T1, α=.84 at T2, and α=.86 at T3; for husbands, reliability was α=.84 at T1, α=.84 at T2, and α=.82 at T3.

#### Social support (T1, T2, T3)

Social support was assessed using the Multidimensional Scale of Perceived Social Support developed by Zimet et al. [31] and translated into Korean by Shin and Lee [32]. It consists of 12 items rated on a 7-point Likert scale, with total scores ranging from 12 to 84. Higher scores indicate greater perceived social support. Reliability was Cronbach’s α=.90 in Shin and Lee [32]. In the present study, reliability for pregnant women was α=.93 at T1, α=.93 at T2, and α=.95 at T3; for husbands, reliability was α=.90 at T1, α=.94 at T2, and α=.95 at T3.

#### Parenting stress (T2, T3)

Parenting stress was assessed using the Korean Parenting Stress Scale developed by Kim and Kang [33]. It consists of 36 items rated on a 5-point Likert scale, with total scores ranging from 36 to 180. Higher scores indicate greater parenting stress. Reliability of the original tool was Cronbach’s α=.88 [33]. In this study, reliability for mothers was α=.86 at T2 and α=.92 at T3; for husbands, reliability was α=.94 at both T2 and T3.

#### Maternal and paternal role confidence (T2, T3)

(1) Maternal role confidence was measured using the Maternal Role Confidence Questionnaire developed by Parker and Zahr [34] and translated by Chae [35]. It consists of 14 items rated on a 5-point Likert scale, with total scores ranging from 14 to 70. Higher scores indicate greater maternal role confidence. Reliability was Cronbach’s α=.78 in Chae [35]. In this study, reliability for mothers was α=.67 at T2 and α=.68 at T3. (2) Paternal role confidence was measured using a 12-item, 5-point Likert scale developed by Pharis [36] and translated by Kim [37]. Total scores range from 12 to 60, with higher scores indicating greater paternal role confidence. Reliability at development was Cronbach’s α=.86 [36]. In this study, reliability for husbands was α=.68 at T2 and α=.67 at T3.

#### Sociodemographic, obstetric, and neonatal characteristics

The first questionnaire included items on participants’ sociodemographic and obstetric characteristics. Sociodemographic characteristics included women’s and husbands’ age, education, family income, and duration of marriage. Obstetric characteristics included high-risk pregnancy diagnosis (gestational hypertensive disorders, GDM, preterm labor, placenta previa, incompetent internal os of the cervix, multiple pregnancy, history of recurrent miscarriage, and fetal growth restriction), hospitalization during pregnancy, and reasons for hospitalization. Neonatal characteristics assessed in the second postpartum questionnaire included type of birth, infant birth weight, gestational age, infant sex, and admission to the neonatal intensive care unit (NICU). Hospitalization during pregnancy was included because hospitalization for high-risk pregnancy complications may affect antenatal health and couples’ QoL.

### Data collection

Data were collected individually using structured questionnaires at three time points: 20 to 32 weeks of gestation (T1), 6 weeks postpartum (T2), and 12 weeks postpartum (T3). T1 was selected because fetal surveillance and intensive management for high-risk pregnancies typically begin during this period [38], when pregnant women and their husbands may experience substantial physical and psychological burden related to high-risk pregnancy. T2 was selected because 6 weeks postpartum is marked by physical recovery and prominent psychosocial changes, including postpartum depression, and has been widely used as a key time point for measuring postpartum QoL in previous studies [38]. T3 was selected because 12 weeks postpartum corresponds to the period recommended by the American College of Obstetricians and Gynecologists for completing comprehensive postpartum care [39], and because follow-up care is particularly emphasized for high-risk mothers with hypertensive disorders, diabetes, and related conditions. In addition, previous studies have shown that several QoL domains improve significantly at 12 to 14 weeks postpartum compared with 6 to 8 weeks postpartum [38].

### Data analysis

Sociodemographic, obstetric, and neonatal characteristics and QoL levels among pregnant women experiencing high-risk pregnancies and their husbands at 20 to 32 weeks of gestation, 6 weeks postpartum, and 12 weeks postpartum were analyzed and compared using frequency analysis and descriptive statistics. Antenatal QoL levels for each partner according to participant characteristics were analyzed using the independent *t*-test and one-way analysis of variance. Because the number of participants varied across time points in this cohort study, changes in QoL by time point and partner were analyzed using a linear mixed model (LMM) that included all available data at each time point. Participants were specified as a random effect (random intercept), and the significance of the time effect was evaluated using a likelihood ratio test. Relationships between QoL and study variables by time point and partner were analyzed using Pearson correlation analysis. Physical and psychosocial factors associated with QoL by time point and partner were analyzed using hierarchical multiple regression analysis.

## Results

### General characteristics of couples with high-risk pregnancies

The sociodemographic characteristics of the 149 couples who participated at T1 showed that the pregnant women had a mean age of 35.97±3.67 years (range, 28 to 46 years), and 98 women (65.7%) were 35 years or older. Most women had a college degree or higher (n=141, 94.6%). The most common monthly family income category was 4 million Korean won or more (n=106, 71.2%). Mean duration of marriage was 3.50±2.37 years, and mean gestational age at enrollment was 28.20±2.52 weeks. Husbands had a mean age of 37.65±4.73 years, and 108 husbands (72.4%) were 35 years or older. Most husbands had a college degree or higher (n=130, 87.2%).

Sixty-five women (43.6%) had been hospitalized because of a high-risk pregnancy. The most frequent high-risk pregnancy diagnoses were GDM and incompetent internal os of the cervix, with 33 cases each (22.1%), followed by preterm labor (31 cases, 20.8%). Other diagnoses included placenta previa and threatened abortion, with 11 cases each (7.4%); premature rupture of membranes nine cases (6.0%); antepartum hemorrhage, seven cases (4.8%); advanced maternal age, seven cases (4.8%); severe preeclampsia, four cases (2.6%); and amniotic-fluid disorders, three cases (2.0%) (Table 1).

Among the 122 participants evaluated at 6 weeks postpartum (T2), 86 (70.5%) had a cesarean birth and 36 (29.5%) had a vaginal birth. Mean infant birth weight was 3.29±2.55 kg, and 21 infants (17.2%) weighed less than 2.5 kg (low birth weight). Gestational age at birth ranged from 31 to 39 weeks; 100 infants (82.0%) were born before 37 weeks of gestation. Infant sex was evenly distributed, with 61 males (50.0%) and 61 females (50.0%). Thirty infants (24.5%) were admitted to the NICU.

Antenatal QoL differed by selected sociodemographic and obstetric characteristics in both pregnant women and their husbands. Among women, QoL differed significantly by education and history of hospitalization. Women with a college degree or higher had significantly higher QoL scores than those with a high school education (F=4.37, *p*=.031), whereas women with a history of hospitalization had significantly lower QoL scores than those without hospitalization (F=3.61, *p*<.001). Among husbands, QoL differed significantly only by education; husbands with a college degree or higher had significantly higher QoL scores than those with a high school education (F=2.66, *p*=.008) (Table 1).

### Quality of life levels and changes in couples with high-risk pregnancies by time point and relationships among study variables

QoL comparisons by partner and time point showed that at T1, women’s overall QoL score was 3.06±0.43, whereas husbands’ score was higher, at 3.39±0.47. At T2 and T3, husbands’ scores remained consistently higher (2.29±0.32 and 2.26±0.33, respectively) than women’s scores (2.05±0.32 and 2.10±0.33, respectively). Compared with antenatal QoL (T1), overall QoL declined during the postpartum period (T2 and T3) in both women and husbands. The time-related change was statistically significant in women (χ^2^=31.83, df=2, *p*<.001) but not in husbands (χ^2^=1.27, df=2, *p*=.530).

In domain-specific analyses by partner and time point, women’s physical health scores decreased from 12.68±1.99 at T1 to 10.91±2.93 at T2 and then partially recovered to 11.15±2.89 at T3 (χ^2^=43.28, df=2, *p*<.001). Psychological health scores increased from 8.95±1.23 at T1 to 12.27±2.67 at T2 and remained stable at 12.24±2.74 at T3 (χ^2^=166.71, df=2, *p*<.001). Social relationships scores were 13.25±2.27 at T1, 11.69±2.80 at T2, and 12.08±2.77 at T3 (χ^2^=39.04, df=2, *p*<.001). Environment scores decreased gradually from 13.81±2.40 at T1 to 13.06±2.45 at T2 and 13.02±2.68 at T3 (χ^2^=16.15, df=2, *p*<.001).

Among husbands, physical health scores increased from 12.65±1.88 at T1 to 14.09±2.49 at T2 and then decreased slightly to 13.73±2.83 at T3 (χ^2^=38.35, df=2, *p*<.001). Psychological health scores increased from 13.56±1.80 at T1 to 14.51±2.33 at T2 and remained relatively stable at 14.20±2.73 at T3 (χ^2^=17.33, df=2, *p*<.001). Social relationships scores decreased slightly from 13.60±2.24 at T1 to 12.86±2.94 at T2 and 12.81±2.95 at T3 (χ^2^=12.72, df=2, *p*=.002). Environment scores remained relatively stable across time points: 14.19±2.42 at T1, 14.31±2.40 at T2, and 13.89±2.79 at T3 (χ^2^=3.39, df=2, *p*=.184). In the psychological health domain, husbands had higher scores than women starting in the antenatal period (13.56 vs. 8.95 at T1) (Table 2, Supplementary Figure 1).

Correlation analyses between QoL and study variables by partner and time point showed that, across all three survey time points, QoL in both pregnant women and husbands was negatively correlated with fatigue and anxiety and positively correlated with social support, the spousal relationship, and attachment. At T2 and T3, parenting stress was negatively correlated with QoL in both mothers and husbands (Table 3).

### Factors associated with quality of life in couples with high-risk pregnancies by time point

Before multiple regression analysis, assumptions of normality, linearity, homoscedasticity, and multicollinearity were assessed. Normality was evaluated using histograms and Q-Q plots, and the Kolmogorov-Smirnov test showed no significant deviation from normality (*p*>.05). Scatter plots of residuals against predicted values showed random distributions, supporting the assumptions of linearity and homoscedasticity. All independent variables had tolerance values of .70 or higher and variance inflation factor values of 1.1 to 2.5, indicating no evidence of multicollinearity. Categorical variables entered into the regression models were dummy-coded. Education for women and husbands was coded as high school or below (reference category, 0) or currently attending college/college graduate or higher (1). History of hospitalization was coded as no hospitalization (reference category, 0) or hospitalization (1).

At T1, the regression model for women’s QoL was significant (F=7.75, *p*<.001) and explained 33.0% of the variance (adjusted R^2^=.33). Factors associated with pregnant women’s QoL were history of hospitalization (β=.21, *p*=.003), fatigue (β=–.23, *p*=.015), anxiety (β=–.44, *p*<.001), social support (β=.29, *p*=.002), and the spousal relationship (β=.24, *p*=.008). The regression model for husbands’ QoL was also significant (F=12.29, *p*<.001) and explained 45.0% of the variance (adjusted R^2^=.45). Factors associated with husbands’ QoL were wives’ history of hospitalization (β=.17, *p*=.019), anxiety (β=–.41, *p*<.001), social support (β=.31, *p*<.001), and the spousal relationship (β=.20, *p*=.018).

At T2, the regression model for women’s QoL was significant (F=23.98, *p*<.001) and explained 60.0% of the variance (adjusted R^2^=.60). Factors associated with mothers’ QoL during this period were anxiety (β=–.38, *p*<.001), parenting stress (β=–.27, *p*<.001), social support (β=.16, *p*=.010), and the spousal relationship (β=.14, *p*=.022). The regression model for husbands’ QoL was also significant (F=20.57, *p*<.001) and explained 63.0% of the variance (adjusted R^2^=.63). Factors associated with husbands’ QoL during this period were wives’ history of hospitalization (β=.14, *p*=.013), anxiety (β=–.42, *p*<.001), parenting stress (β=–.22, *p*=.007), social support (β=.19, *p*=.016), and the spousal relationship (β=.17, *p*=.016).

At T3, the regression model for women’s QoL was significant (F=25.64, *p*<.001) and explained 66.0% of the variance (adjusted R^2^=.66). Factors associated with mothers’ QoL during this period were fatigue (β=–.18, *p*=.016), anxiety (β=–.43, *p*=.001), and social support (β=.30, *p*<.001). The regression model for husbands’ QoL was also significant (F=20.15, *p*<.001) and explained 61.0% of the variance (adjusted R^2^=.61). Factors associated with husbands’ QoL during this period were fatigue (β=–.18, *p*=.015), anxiety (β=–.51, *p*<.001), parenting stress (β=–.31, *p*=.002), and social support (β=.12, *p*=.045) (Table 4).

## Discussion

This study examined changes in QoL among pregnant women experiencing high-risk pregnancies and their husbands from the antenatal period to 6 and 12 weeks postpartum, as well as factors associated with QoL at each time point. Anxiety was consistently and negatively associated with QoL in both partners across all time points, whereas social support was consistently and positively associated with QoL throughout the study period. These findings indicate that high-risk pregnancy is not a discrete stressor limited to pregnancy but a continuing psychosocial context that extends into the postpartum period. The antenatal findings are consistent with previous work showing that psychosocial factors, including anxiety and social support, are associated with health-related QoL among pregnant women [11]. The postpartum findings also support a systematic review reporting that perceived stress is closely related to postpartum depressive symptoms and maternal mental health [19]. QoL should therefore be understood not as a static state but as a trajectory that changes during the transition to parenthood. This study contributes by showing that stressors and resistance resources differ by time point in both pregnant women and husbands and by describing partner-specific QoL changes in couples experiencing high-risk pregnancy.

According to Antonovsky’s salutogenic theory [18], health and well-being in stressful situations are shaped by GRRs and SOC. Using this framework, the present study empirically supported the premise that anxiety-related stressors threaten QoL, whereas resistance resources such as social support help maintain or improve health. Resistance resources may help individuals understand, manage, and find meaning in their circumstances, even amid the continuing uncertainty of high-risk pregnancy [17,19]. Improving QoL in couples experiencing high-risk pregnancy therefore requires a salutogenic strategy that strengthens positive resources, including social support, role confidence, and the spousal relationship, rather than focusing only on stress reduction. Such interventions should address anxiety while also expanding multidimensional protective resources that support psychological and emotional stability and adaptation in both partners.

Among pregnant women experiencing high-risk pregnancies, anxiety was negatively associated with QoL at all time points, and this association persisted across the antenatal and postpartum periods. By time point, a high-risk pregnancy diagnosis may create continuing uncertainty about pregnancy maintenance and fetal health. This uncertainty may intensify psychological anxiety as well as physical fatigue, both of which were identified as negative factors for QoL. These findings are consistent with studies reporting that physical symptoms and activity restrictions related to high-risk pregnancy shape QoL experiences [9,12] and that physical and psychological vulnerability in high-risk pregnant women is closely associated with lower QoL [40,41]. In contrast, the finding that the spousal relationship and social support were associated with QoL is consistent with previous research describing their protective role in reducing antenatal anxiety and supporting emotional well-being [10,19,30].

At 6 weeks postpartum, factors associated with mothers’ QoL differed from those identified during pregnancy. Parenting stress related to parental role performance, together with anxiety, was negatively associated with QoL, whereas physical burden was no longer the central factor. This pattern suggests that the psychological burden of early infant care and adaptation to the maternal role becomes a central context for QoL in the early postpartum period, consistent with previous studies [12,19]. The diminished influence of physical fatigue, which was important during pregnancy, is also consistent with reports that high-risk pregnancy-related fatigue lessens after childbirth and that parenting-related stress becomes relatively more important as physical recovery progresses [10,42]. The finding that the spousal relationship was positively associated with mothers’ QoL also corresponds with a study reporting a sustained positive correlation between the spousal relationship and QoL measured at 6 weeks postpartum [42]. Thus, during the transition to parenthood, a stable and positive spousal relationship may facilitate parental role adaptation and improve QoL in both partners.

At 12 weeks postpartum, factors associated with mothers’ QoL again centered on fatigue, anxiety, and social support. This finding supports previous reports [18] suggesting that even after the initial postpartum adaptation period, physical and psychological status and social support remain central to QoL. The sustained influence of social support from pregnancy through 12 weeks postpartum is also consistent with literature emphasizing the importance of practical and emotional support from family and society as postpartum childcare demands increase [19,31].

Among husbands, QoL was also associated with anxiety, social support, and the spousal relationship, but the pattern of associated factors varied more by time point than it did among mothers. During the antenatal period, anxiety was the main negative factor, whereas social support and the spousal relationship were positive factors for QoL. This finding is consistent with previous studies suggesting that, in high-risk pregnancy, husbands’ QoL is shaped not only by their own emotional state but also by the relational context [16,19]. Physical factors were not significantly associated with husbands’ QoL during pregnancy, likely because the physical risks and burdens of high-risk pregnancy are borne primarily by the pregnant women [16].

At 6 weeks postpartum, social support and the spousal relationship were positive factors for husbands’ QoL, whereas anxiety and parenting stress related to paternal role performance were negative factors. This finding suggests that in the early postpartum period, husbands are transitioning beyond a supportive role in housework and childcare into active participation in infant care and adaptation to a new parental role. It is consistent with previous studies reporting an association between paternal role adaptation and QoL [5,16,43]. It also parallels the finding that mothers’ spousal relationship was a positive factor for QoL and aligns with research showing that the spousal relationship and QoL measured at 6 weeks postpartum remained influential at 6 months postpartum [41]. During the transition to parenthood, more positive mutual perceptions of the spousal relationship may therefore be associated with better health-related QoL in both partners.

At 12 weeks postpartum, husbands’ QoL decreased slightly compared with 6 weeks postpartum, but the magnitude of change was small, indicating a relatively stable pattern. In the regression analysis, however, the spousal relationship, which had been a significant protective factor for husbands’ QoL through 6 weeks postpartum, was no longer significant at 12 weeks postpartum; paternal role confidence also was not significant. These findings suggest that the influence of relational resources supporting husbands’ QoL may weaken after the early postpartum period. On the basis of these data alone, it is difficult to determine whether this change is a temporary pattern specific to 12 weeks postpartum or whether other psychosocial resources become more important during paternal role adaptation. Future research should examine husbands’ postpartum QoL more deeply by extending repeated-measure designs or incorporating qualitative methods [16].

In summary, anxiety and social support were the key factors common to both pregnant women and husbands, but additional factors differed by time point and partner. Pregnant women’s QoL was mainly associated with psychological stability and social support, whereas husbands’ QoL was also associated with relational factors, such as the spousal relationship, through the early postpartum period. By 12 weeks postpartum, this relational influence appeared to weaken, and husbands’ QoL showed a pattern more similar to that of pregnant women, centering on anxiety and social support. These findings suggest that husbands may also be a psychosocially vulnerable group during high-risk pregnancy and the postpartum transition. They support integrated nursing interventions that move beyond pregnant-woman-centered care, include couples as the unit of intervention, and address time-specific factors from pregnancy through the postpartum period [16,19].

This study provides a theoretical contribution by applying salutogenic theory in a longitudinal analytical framework to explain QoL changes in couples experiencing high-risk pregnancy. The couple-based design, which analyzed pregnant women and their husbands simultaneously, addresses limitations of previous mother-centered research and extends maternal nursing perspectives and practice toward the family unit. By following changes from pregnancy through the postpartum period, the study also provides methodological evidence for longitudinal survey research on the transition to parenthood.

From a practice perspective, this study identified key factors associated with QoL at antenatal and postpartum time points and provides foundational evidence for developing time-specific, tailored nursing interventions for couples experiencing high-risk pregnancy. The findings strengthen the practical basis for family-centered nursing approaches that include husbands as well as pregnant women and identify factors that nurses can consider when designing interventions in clinical settings.

At the social level, this study provides empirical evidence that high-risk pregnancy affects QoL and parental adaptation in families transitioning to parenthood. The findings highlight the need to improve social awareness and develop psychosocial support systems for these families. They may also support expansion of family support systems, including community-based education programs and self-help groups.

This study has several limitations. First, the sample size was limited, and variables were measured using self-report questionnaires. Second, because participants were recruited through an online Mom cafe, sample representativeness and selection bias should be considered, and the findings should be generalized with caution. Future studies should improve representativeness through multicenter designs involving diverse medical institutions. Third, some scales had relatively low internal consistency, and the effects of these variables should be interpreted conservatively. In addition, the pregnancy stress tool was originally developed and validated only for pregnant women and had not been validated for husbands, but it was applied to husbands without item modification. Other tools, including anxiety and fatigue measures, were also administered to both partners using the same items, which may not have fully captured husbands’ experiences. Fourth, although repeated-measure longitudinal data were collected at three time points, the LMM accounted for the correlation structure of repeated measures, but attrition during follow-up was not addressed through formal missing-data or sensitivity analyses (e.g., multiple imputation). Future studies should examine QoL trajectories in high-risk pregnancy families more rigorously using longitudinal methods, such as multiple imputation or generalized estimating equations, in addition to linear mixed-effects models. Qualitative studies should also be conducted to examine the experiences of pregnant women and husbands in greater depth and to further evaluate the applicability of salutogenic theory and family-centered nursing interventions.

In conclusion, this longitudinal study of pregnant women experiencing high-risk pregnancies and their husbands found that QoL declined in both partners during the transition from pregnancy to parenthood, and factors associated with QoL differed by time point and partner. Anxiety and social support were common factors associated with QoL in both pregnant women and husbands from pregnancy through 12 weeks postpartum. Pregnancy-related burdens were relatively more important during the antenatal period, whereas factors related to parental role performance became more important during the postpartum period. These findings suggest that QoL among couples experiencing high-risk pregnancy is not fixed but is shaped by the interaction of time-varying stressors and psychosocial resources. Future research should treat the couple as the unit of analysis and examine how mutual stressors and resources intersect and interact.

## Figures and Tables

**Figure 1.**
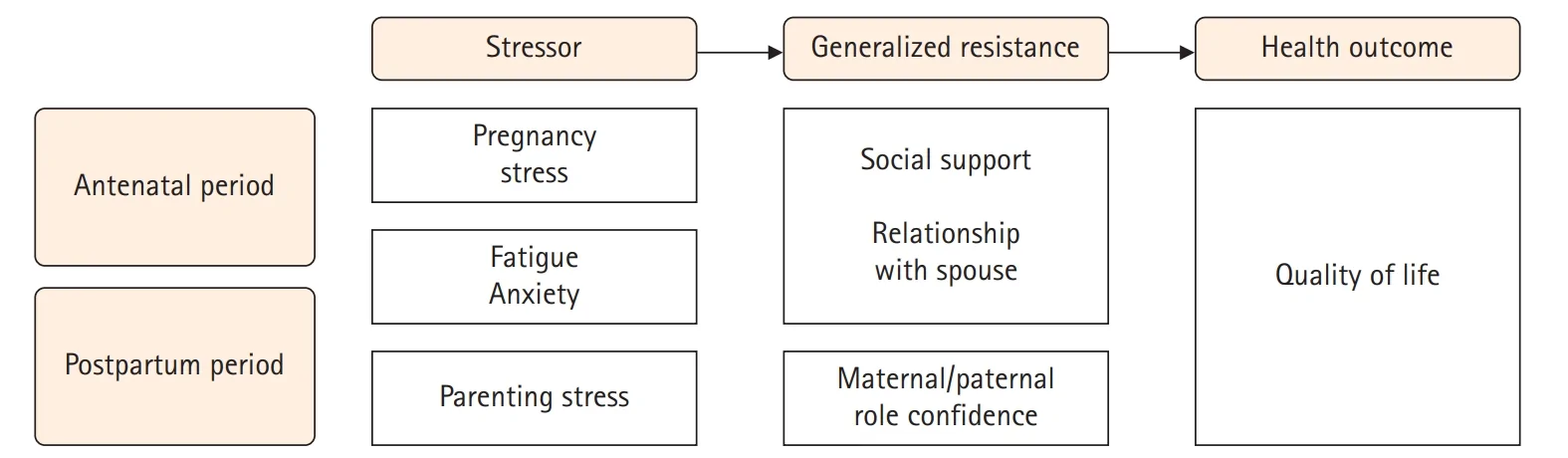
Theoretical model of quality of life across time points in high-risk pregnancy couples based on the Salutogenic theory.

**Figure 2.**
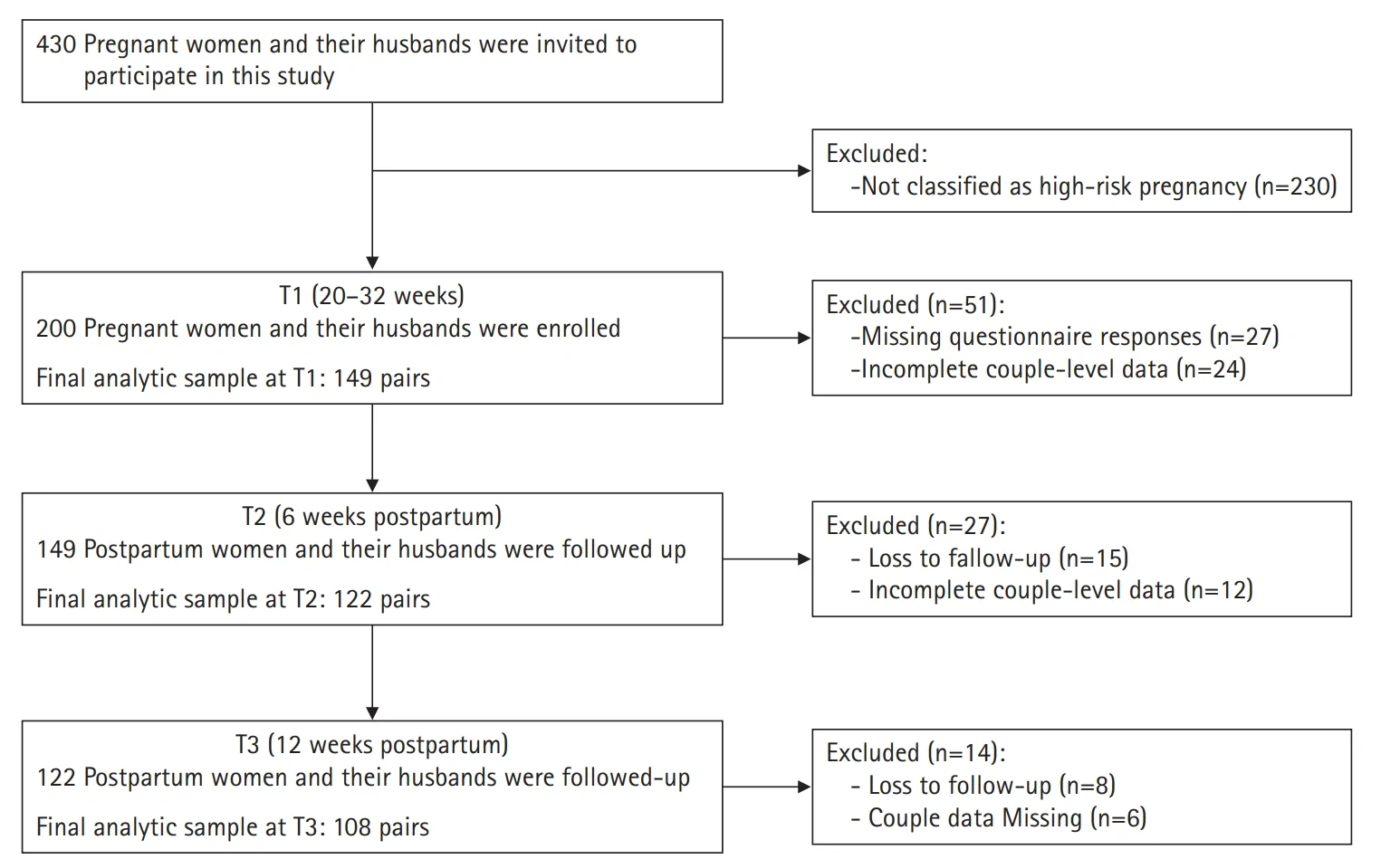
Flowchart of the study cohort. T1: 20–32 weeks of pregnancy; T2: 6 weeks postpartum; T3: 12 weeks postpartum.

**Table 1.** Differences in quality of life between pregnant women and their husbands by participant characteristics (N=149)

Variables	Category	n (%)	Mean±SD	Quality of life			
				Women	t/F (*p*)	Husbands	t/F (*p*)
Women’s characteristics							
Women’s age (year)	≤34	51 (34.3)	35.97±3.67	3.04±0.38	0.86 (.475)	-	-
	≥35	98 (65.7)		3.09±0.43		-	
Women’s education	High school	8 (5.4)		3.21±0.45	4.37 (.031)	-	-
	≥College	141 (94.6)		3.44±0.46		-	
Family income (×10,000 KRW)	<200	2 (1.3)		3.19±0.39	2.98 (.054)	3.44±0.68	1.35 (.261)
	200–399	41 (27.5)		2.94±0.45		3.32±0.47	
	≥400	106 (71.2)		3.12±0.39		3.46±0.45	
Duration of marriage (year)	-	-	3.50±2.37	3.07±0.49	0.84 (.731)	3.42±0.46	1.35 (.148)
Gestational period at enrollment (wk)	-	-	28.2±2.52	-	-	-	-
History of hospitalization	Yes	65 (43.6)		2.93±0.41	3.61 (<.001)	3.36±0.46	0.08 (.079)
	No	84 (56.4)		3.18±0.38		3.47±0.45	
Diagnosis of high-risk pregnancy							
Diagnosis of high-risk pregnancy	GDM	33 (22.1)		3.13±0.47	0.80 (.423)	3.37±0.54	0.27 (.782)
	IIOC	33 (22.1)		2.87±0.35	2.26 (.031)	3.29±0.45	1.12 (.262)
	Preterm labor	31 (20.8)		2.99±0.45	1.04 (.302)	3.45±0.41	0.72 (.473)
	Placenta previa	11 (7.4)		3.15±0.35	0.51 (.608)	3.51±0.16	0.75 (.454)
	Threatened abortion	11 (7.4)		3.01±0.40	0.38 (.705)	3.16±0.40	1.43 (.153)
	PROM	9 (6.0)		2.69±0.19	4.49 (.002)	3.21±0.31	0.96 (.336)
	Antepartum hemorrhage	7 (4.8)		2.99±0.35	0.41 (.677)	3.28±0.37	0.51 (.606)
	Advanced maternal age	7 (4.8)		3.09±0.38	0.69 (.491)	3.42±0.47	1.02 (.309)
	Severe preeclampsia	4 (2.6)		3.00±0.45	0.33 (.736)	3.05±0.32	1.50 (.135)
	Amniotic fluid disorders	3 (2.0)		2.05±0.42	0.43 (.332)	2.11±0.18	1.30 (.195)
Obstetric and neonatal characteristics^†^							
Type of birth	Vaginal birth	36 (29.5)		3.08±0.42	2.38 (.022)	3.42±0.46	0.08 (.709)
	Cesarean birth	86 (70.5)		3.10±0.08		3.46±0.48	
Infant’s birth weight (kg)	<2.5	21 (17.2)		2.14±0.27	0.90 (.343)	2.36±0.33	0.88 (.386)
	≥2.5	101 (82.8)		2.06±0.33		2.29±0.33	
Gestational age (wk)	≥37	22 (18.0)		2.14±0.30	0.96 (.171)	2.33±0.33	0.83 (.383)
	<37	100 (82.0)		2.06±0.33		2.29±0.33	
Infant’s sex	Male	61 (50.0)		2.07±0.34	0.60 (.776)	2.28±0.35	0.97 (.205)
	Female	61 (50.0)		2.08±0.31		2.33±0.31	
NICU admission of infants	Yes	30 (24.5)		2.10±0.34	0.33 (.668)	2.35±0.31	0.01 (.326)
	No	92 (75.5)		2.07±0.32		2.29±0.33	
Husbands’ characteristics							
Husbands’ age (year)	≤34	41 (27.6)	37.65±4.73	-	-	3.46±0.45	0.55 (0.59)
	≥35	108 (72.4)		-		3.41±0.46	
Husbands’ education	High school	19 (12.8)		-	-	3.17±0.38	2.66 (.008)
	≥College	130 (87.2)		-		3.46±0.46	

GDM: Gestational diabetes mellitus; IIOC: incompetent internal os of cervix; KRW: Korean won (1 USD=approximately 1,500 KRW); NICU: neonatal intensive care unit; PROM: premature rupture of membranes; QoL: quality of life.

† Obstetric and neonatal characteristics were assessed at 6 weeks postpartum; n=122.

**Table 2.** Comparison of QoL between women and their husbands across time points

Domains of QoL		Mean±SD			χ^2^ (df)^†^	*p*
		T1 (n=149)	T2 (n=122)	T3 (n=108)		
Women	Total	3.06±0.43	2.05±0.32	2.10±0.33	31.83 (2)	<.001
	Physical health	12.68±1.99	10.91±2.93	11.15±2.89	43.28 (2)	<.001
	Psychological health	8.95±1.23	12.27±2.67	12.24±2.74	166.71 (2)	<.001
	Social relationships	13.25±2.27	11.69±2.80	12.08±2.77	39.04 (2)	<.001
	Environment	13.81±2.40	13.06±2.45	13.02±2.68	16.15 (2)	<.001
Husbands	Total	3.39±0.47	2.29±0.32	2.26±0.33	1.27 (2)	.530
	Physical health	12.65±1.88	14.09±2.49	13.73±2.83	38.35 (2)	<.001
	Psychological health	13.56±1.80	14.51±2.33	14.20±2.73	17.33 (2)	<.001
	Social relationships	13.60±2.24	12.86±2.94	12.81±2.95	12.72 (2)	.002
	Environment	14.19±2.42	14.31±2.40	13.89±2.79	3.39 (2)	.184

QoL: Quality of life; T1: 20–32 weeks of pregnancy; T2: 6 weeks postpartum; T3: 12 weeks postpartum.

† The time effect was tested using a linear mixed model with a random intercept for participant and a likelihood ratio test; this approach incorporated all available data at each time point rather than restricting the analysis to participants with complete data across all three time points.

**Table 3.** Relationships between QoL and related variables at T1, T2, and T3 in women and their husbands

Time point	Number	r (*p*)					
		Fatigue	Anxiety	Social support	Relationship with spouse	Attachment	Parenting stress
Women							
T1	149	–.25 (.001)	–.58 (<.001)	.37 (.001)	.40 (<.001)	.23 (.004)	–
T2	122	–.35 (<.001)	–.62 (<.001)	.36 (<.001)	.29 (<.001)	.26 (<.001)	–.57 (<.001)
T3	108	–.46 (<.001)	–.69 (<.001)	.30 (<.001)	.42 (<.001)	.49 (<.001)	–.59 (<.001)
Husbands				r(*p*)			
T1	149	–.29 (<.001)	–.62 (<.001)	.50 (<.001)	.17 (.029)	.42 (<.001)	–
T2	122	–.25 (.005)	–.67 (<.001)	.40 (<.001)	.59 (<.001)	.50 (<.001)	–.58 (<.001)
T3	108	–.23 (.017)	–.67 (<.001)	.26 (.005)	.49 (<.001)	.45 (<.001)	–.63 (<.001)

QoL: Quality of life; T1: 20–32 weeks of pregnancy; T2: 6 weeks postpartum; T3: 12 weeks postpartum.

**Table 4.** Factors associated with quality of life in women and their husbands at T1, T2, and T3

Variable	β (*p*)																	
	T1 (n=149)						T2 (n=122)						T3 (n=108)					
	Women			Husbands			Women			Husbands			Women			Husbands		
	Model 1	Model 2	Model 3	Model 1	Model 2	Model 3	Model 1	Model 2	Model 3	Model 1	Model 2	Model 3	Model 1	Model 2	Model 3	Model 1	Model 2	Model 3
Women’s education^†^	.13 (.157)	.17 (.059)	.04 (.536)	.06 (.520)	.12 (.143)	.06 (.373)	.05 (.592)	.10 (.103)	.08 (.156)	.01 (.656)	.03 (.609)	.01 (.941)	.06 (.552)	.08 (.226)	.11 (.059)	.04 (.692)	.02 (.652)	–.01 (.785)
Husband’s education^†^	.02 (.881)	.02 (.827)	.06 (.347)	.03 (.725)	.09 (.270)	.64 (.415)	.01 (.842)	.01 (.956)	.02 (.968)	.10 (.287)	.03 (.580)	.05 (.427)	.06 (.518)	.11 (.078)	.09 (.109)	.03 (.727)	.07 (.278)	.07 (.221)
History of hospitalization^†^	.27 (.006)	.27 (.003)	.21 (.003)	.19 (.049)	.23 (.007)	.17 (.019)	.22 (.015)	.09 (.142)	.09 (.123)	.24 (.008)	.16 (.007)	.14 (.013)	.05 (.561)	.04 (.541)	.05 (.324)	–.05 (.559)	–.07 (.241)	–.05 (.413)
Fatigue		–.19 (.075)	–.23 (.015)		.04 (.614)	–.10 (.175)		–.02 (.293)	–.12 (.072)		–.10 (.012)	–.07 (.273)		–.14 (.084)	–.18 (.016)		–.19 (.005)	–.18 (.015)
Anxiety		–.53 (<.001)	–.44 (<.001)		–.58 (<.001)	–.41 (<.001)		–.47 (<.001)	–.38 (<.001)		–.59 (<.001)	–.42 (<.001)		–.51 (<.001)	–.43 (.001)		–.57 (<.001)	–.51 (<.001)
Stress^‡^		–.20 (.057)	–.11 (.225)		–.04 (.628)	–.06 (.461)		–.31 (<.001)	–.27 (<.001)		–.24 (<.001)	–.22 (.007)		–.20 (.029)	–.14 (.096)		–.37 (<.001)	–.31 (<.001)
Social support			.29 (.002)			.31 (<.001)			.16 (.010)			.19 (.016)			.30 (<.001)			.12 (.045)
Relationship with spouse			.24 (.008)			.20 (.018)			.14 (.022)			.17 (.016)			.07 (.221)			.11 (.123)
Role confidence			–			–			.04 (.578)			.11 (.073)			.01 (.874)			–.04 (.692)
F (*p*)	3.84 (.012)	4.21 (<.001)	7.75 (<.001)	2.76 (.044)	8.38 (<.001)	12.29 (<.001)	2.45 (.067)	29.32 (<.001)	23.98 (<.001)	2.96 (.035)	20.85 (<.001)	20.57 (<.001)	2.66 (.052)	25.54 (<.001)	25.64 (<.001)	0.22 (.882)	27.94 (<.001)	20.15 (<.001)
Adjusted R^2^	.07	.15	.33	.03	.29	.45	.03	.54	.60	.04	.58	.63	.04	.56	.66	.02	.59	.61

T1: 20–32 weeks of pregnancy; T2: 6 weeks postpartum; T3: 12 weeks postpartum. Model 1: women’s education, husband’s education, and history of hospitalization; Model 2: Model 1 plus fatigue, anxiety, and stress; Model 3: Model 2 plus social support and relationship with spouse, with role confidence additionally included at T2 and T3.

† Reference categories: women’s/husbands’ education (reference=high school); history of hospitalization (reference=no).

‡ Stress refers to pregnancy stress at T1 and parenting stress at T2 and T3.

## Supplementary materials

Further details on supplementary materials are presented online (available at https://doi.org/10.4069/whn.2026.08.21).

Supplementary Figure 1. Trends in quality of life domains across time points for women and husbands (mean±SD). T1: 20–32 weeks of pregnancy; T2: 6 weeks postpartum; T3: 12 weeks postpartum.
