# Supplementary material for: Perinatal quality-of-life trajectories and determinants among high-risk pregnant women and their husbands: a prospective cohort study
Source: Womens Health Nurs. 2026 Sep 29;32(3):218–32. doi: 10.4069/whn.2026.08.21 (PMC13654683; doi:10.4069/whn.2026.08.21)
Supplement: Supplementary file 1 [file whn-2026-08-21-Supplementary-Figure-1.pdf]

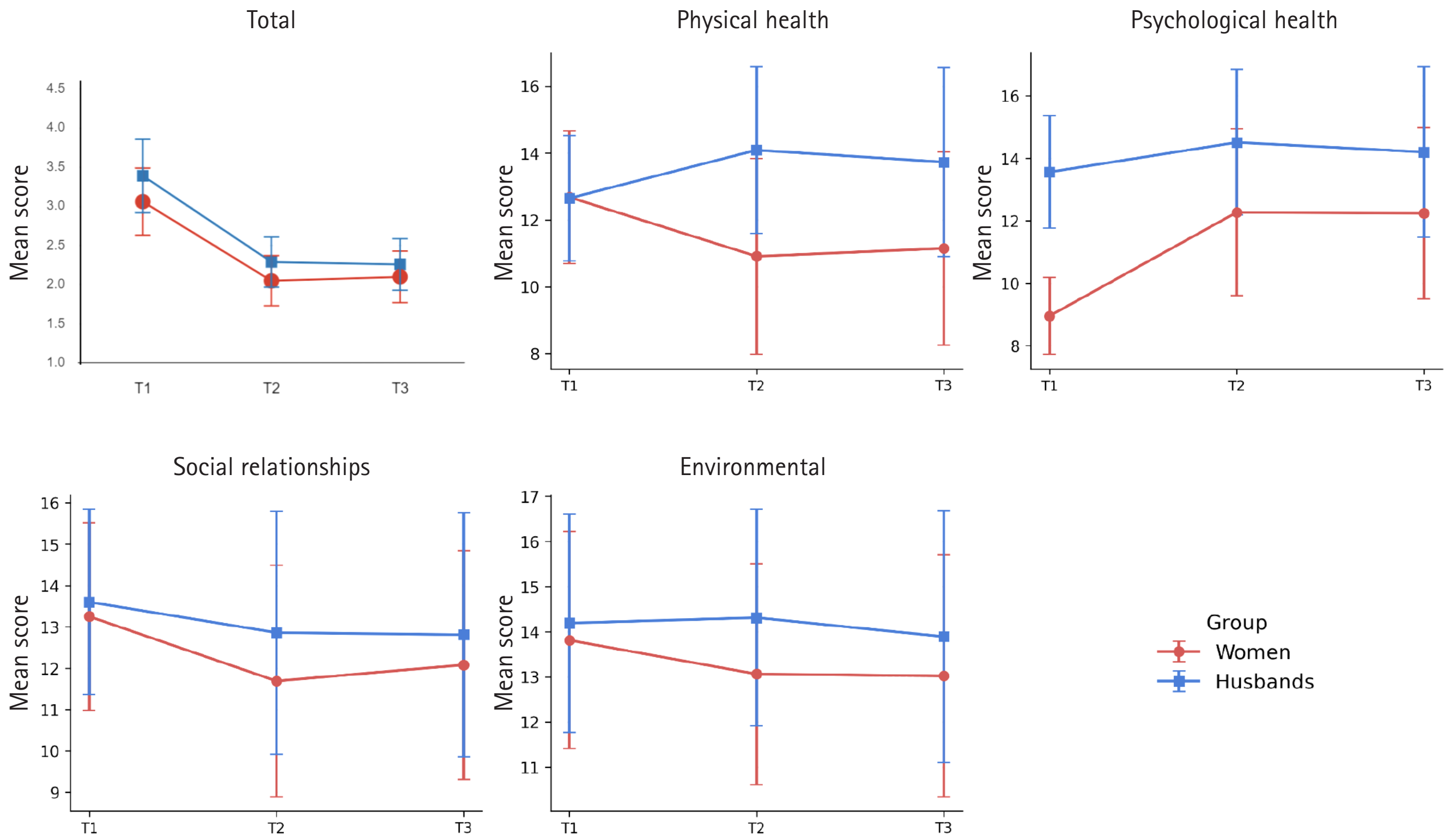

Supplementary Figure 1. Trends in quality of life domains across time points for women and husbands (mean±SD). T1: 20–32 weeks of pregnancy; T2: 6 weeks postpartum; T3: 12 weeks postpartum.
